# Pathologic Findings of Chronic PML-IRIS in a Patient with Prolonged PML Survival Following Natalizumab Treatment

**DOI:** 10.1177/2324709617734248

**Published:** 2017-09-27

**Authors:** Mai Himedan, Sandra Camelo-Piragua, Elizabeth A. Mills, Avneesh Gupta, Rany Aburashed, Yang Mao-Draayer

**Affiliations:** 1 University of Michigan, Ann Arbor, MI, USA; 2 Michigan State University, East Lansing, MI, USA

**Keywords:** progressive multifocal leukoencephalopathy, multiple sclerosis, inflammation, natalizumab

## Abstract

Immune reconstitution inflammatory syndrome (IRIS) is a common complication during treatment for natalizumab-associated progressive multifocal leukoencephalopathy (PML). Although severe IRIS can result in acute worsening of disability and is associated with poor prognosis, effective immune reconstitution may account for the high survival rate of this cohort of PML patients. We present pathological evidence of chronic IRIS 3.5 years after diagnosis with natalizumab-associated PML. Our case showed that the IRIS initially developed after plasma exchange therapy and resolved clinically and radiologically following a combination treatment with corticosteroids, maraviroc, and cidofovir. Autopsy 3.5 years later revealed evidence of grey-white matter junction demyelinating lesions characteristic of PML and perivascular leukocyte infiltrates predominated by CD8^+^ T-lymphocytes, and polymerase chain reaction analysis demonstrated the presence of JC viral DNA in this tissue, indicative of persistent PML-IRIS. While clinical symptoms of PML-IRIS typically stabilize within 6 months, our case report suggests that prolonged low-grade inflammation may persist in some patients. Better assays are needed to determine the prevalence of prolonged low-grade IRIS among PML survivors.

## Introduction

Progressive multifocal leukoencephalopathy (PML) is a rare progressive demyelinating disease caused by a mutated form of the John Cunningham virus (JCV) that often results in severe debilitation or death. JCV infection is common, such that 70% to 90% of people have antibodies by adulthood,^1^ but it typically only results in PML in those who are immunocompromised due to human immunodeficiency virus infection, hematological malignancies, or immunomodulating therapies.^2^ One of the immunomodulating therapies associated with PML is natalizumab (Tysabri, Biogen-Idec Inc, Cambridge, MA), a monoclonal antibody used for the treatment of multiple sclerosis (MS).^3^ Natalizumab prevents the passage of a subset of inflammatory cells across the brain endothelium by targeting α4 integrin.^4^

Recovery of immune function in the central nervous system (CNS), through the cessation of natalizumab and clearance through plasma exchange (PLEX), has been the mainstay of treatment in the context of natalizumab-associated PML.^5^ Immune reconstitution inflammatory syndrome (IRIS) often occurs following PLEX, with a prevalence of approximately 70% in patients with natalizumab-associated PML.^6^ High-dose corticosteroids have traditionally been used to dampen the inflammation of IRIS in order to avoid the serious complications of a reconstituted immune system,^7^ but they can also impede the body’s ability to fight JCV.^5^ More recently, maraviroc, a chemokine receptor 5 (CCR5) antagonist initially approved for the treatment of human immunodeficiency virus, has been used to manage PML-IRIS, due to the implication of CCR5^+^ T-cells in JCV replication.^8,9^ While there are currently no consistently effective anti-JCV treatments, several therapeutic agents have been used in PML patients with varying degrees of success. The antiviral cidofovir is active against the polyomavirus SV40,^10^ inhibits JCV replication in cell culture,^11^ and has been described as a useful adjunct in several PML case studies.^12^ It has also been suggested that immunocompetent patients prone to PML-IRIS are more likely to benefit from cidofovir,^13^ indicating a possible role in the mitigation of IRIS, rather than a direct effect on JCV.^12^ Since JCV infection of glial cells involves serotonergic 5HT2A receptors, medications targeting these receptors have also been proposed as potential adjuncts.^14^ Use of the atypical antidepressant mirtazapine has been associated with clinical improvement in some PML patients, with earlier treatment related to better outcomes.^15^

Nonsurvivors with natalizumab-associated PML-IRIS typically die within 6 months.^16^ The longest reported active IRIS on biopsy has been 9 months, which occurred in a patient with a fatal outcome.^17^ Here we present the clinical, radiological, and pathological features of a case of PML occurring during natalizumab use and subsequent IRIS treated with maraviroc that led to prolonged survival with later autopsy findings indicating active IRIS associated inflammation 3.5 years after initial diagnosis.

## Case Report

**Figure 1.**
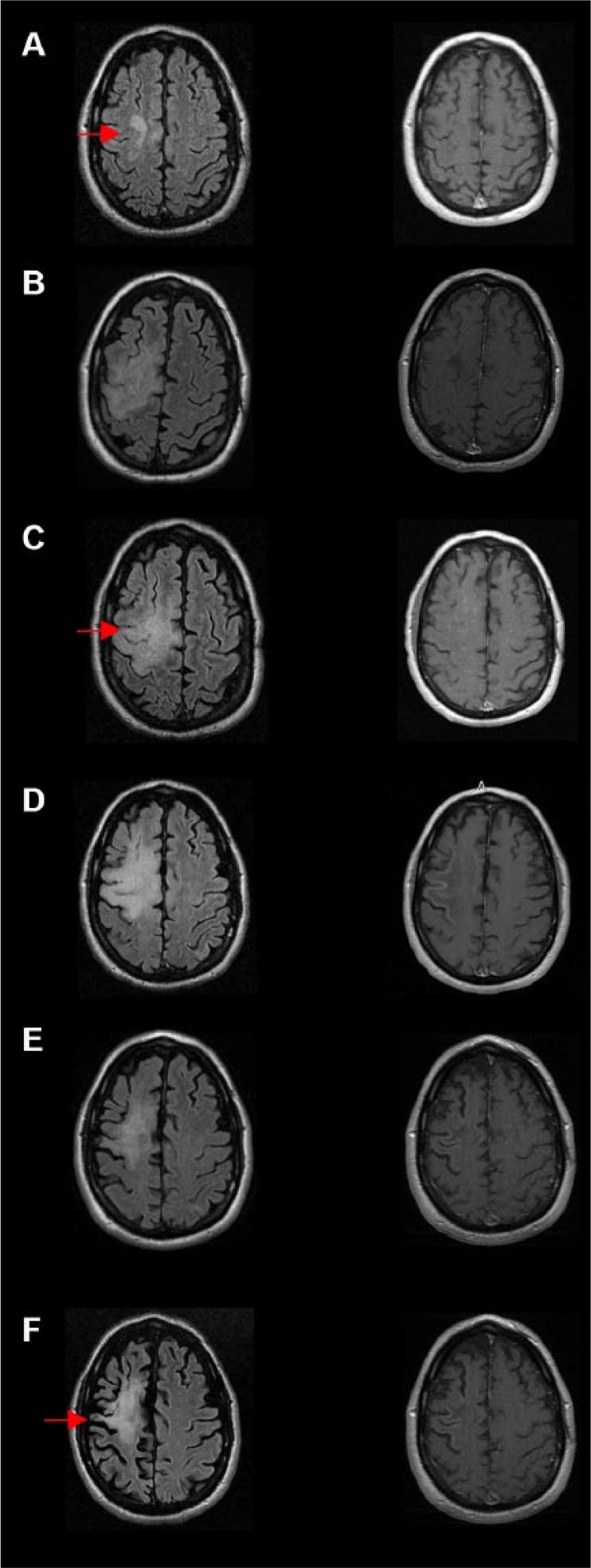
MRI progression of progressive multifocal leukoencephalopathy (PML) and immune reconstitution inflammatory syndrome (IRIS). (A) Initial imaging suspicious for PML. A new confluent hyperintensity was shown in the right superior frontal cortical and subcortical white matter region (arrow). This was confirmed to be PML with CSF JCV studies. (B) The patient presented 2 months later with worsened symptoms, and MRI showed worsened and enlargement of the lesion on T2 FLAIR (arrow; left column B, C, D), with patchy enhancement on T1 post contrast (right column B, C, D). The worsening clinical picture represents acute IRIS. Images C, D, and E show the progression of acute PML-IRIS lesions 2.5 months, 4 months, and 7 months from the diagnosis. (F) Decreased hyperintensity with increasingly apparent brain atrophy (arrow) was seen 14 months after the diagnosis. Left-side column: T2 FLAIR sequence; Right-side column: T1-post contrast.

The patient was a 49-year-old man diagnosed with relapsing-remitting MS in 2003, previously treated with interferon beta-1a (IFNβ1-a, Rebif) and mitoxantrone (Novantrone), and who began treatment with natalizumab in 2009 (300 mg intravenous infusion every 4 weeks) for 3.5 years without relapse. Serum JCV antibodies were detected in September 2011, but the patient elected to continue treatment with natalizumab. He subsequently tested negative for JCV DNA in cerebrospinal fluid (CSF) using polymerase chain reaction (PCR) in November 2011. In May 2013, he presented with 2 weeks of worsening balance and left-sided weakness involving arm, leg, and face. Magnetic resonance imaging (MRI) of the brain with T2 FLAIR sequence revealed a new confluent area of hyperintense signal in the right superior frontal cortical and subcortical white matter region without edema or enhancement (Figure 1A). PML was suspected and natalizumab was discontinued 2 weeks after the onset of symptoms. CSF was sent to Quest Diagnostics in May 2013 for JCV PCR analysis, and then confirmed by an assay at the National Institutes of Health (NIH), which is more sensitive and has a lower cutoff. The copy number of JCV DNA in the CSF was found to be increased at 545 (normal <50, Quest Diagnostics-focus) and 114 (normal <10, NIH/National Institute of Neurological Disorders and Stroke), while serum tested negative for JCV DNA. The patient was begun on plasmapheresis for 5 days, 400 mg/kg intravenous immunoglobulin (IVIG) daily for 5 days, cidofovir for 1 day, and the corticosteroid solumedrol for 5 days. IVIG 400 mg/kg was repeated at day 14. Cidofovir once weekly and IV solumedrol 5 days weekly was repeated for 2 weeks.

In late June 2013, he was readmitted for worsening extremity weakness more pronounced on the left side. MRI showed interval worsening of T2 signals in the right frontal region and interval development of a patchy area of enhancement and punctate enhancement in the left cerebral hemisphere and in the posterior fossa (Figure 1B and C). The worsening clinical picture and MRI features represent acute IRIS. He was administered maraviroc 150 mg BID (bis in die), solumedrol 500 mg BID for 2 days, and mirtazapine, as well as restarted on plasmapheresis and IVIG. To reduce the high risk of seizures with natalizumab-associated PML-IRIS,^18^ he also received the antiepileptic levetiracetam. In July 2013, a repeat CSF JCV PCR test was ordered and showed a value down below the level of detection. He continued outpatient treatment with cidofovir and maraviroc. From December 2013 through March 2014, he was admitted to the hospital several times for infectious symptoms including nausea, vomiting, and fever. Initially, he was diagnosed with *Enterococcus faecalis* based urosepsis, then with a urinary tract infection caused by *Morganella*, and finally was found to have fungemia with *Aspergillus* and *Candida albicans*. He was effectively treated with voriconazole, vancomycin, and piperacillin-tazobactam, and his treatment with maraviroc was subsequently stopped. His neurological deficits stabilized with residual minimal left facial weakness, 2+ strength of the left ankle and toes, but no movement in left knee or hip in November 2013. Six months after his diagnosis of PML, he was begun on Rebif subcutaneous 44 µg 3×/week. He was stable until his death from urosepsis in January 2017. The progression of his PML signal abnormalities were followed by subsequent MRIs; persistent PML-IRIS lesion was seen 4 months after the diagnosis (Figure 1D) and gradually resolved 7 months later (Figure 1E). Fourteen months after PML diagnosis, MRI showed decreased FLAIR hyperintensity and apparent brain atrophy.

### Brain Autopsy Findings

A brain autopsy consent was obtained and brain removal and examination was performed at the University of Michigan. The left hemi-brain was frozen and banked per Michigan Alzheimer’s Disease Core Center banking protocol. The right hemi-brain was fixed in 10% formalin for neuropathologic examination. Coronal sections revealed a large white matter–based, friable, discolored, pitted, soft, and partially cavitary lesion (9 × 3 × 3 cm) of the frontoparietal lobe, which corresponded to the lesion identified radiologically in May 2013, when CSF PML copy numbers were elevated. In addition, there were other demyelinating lesions involving genu and selenium of corpus callosum, posterior limb of internal capsule, posterior operculum, cerebellum, optic nerve, insula, and brain stem. All sections containing demyelinating plaques were stained with anti-SV40 (Cell Marque-Sigma-Aldrich, Clone MRQ4, pre-diluted, Rocklin, CA), but there was no evidence of active polyomavirus infection by this immunohistochemical stain.

In addition to the severely destructive chronic demyelinated plaque of the frontoparietal region, other demyelinating lesions included microscopic “salt on ice” irregular demyelination in grey-white matter junction (PML lesions), larger more confluent plaques with periventricular localization (classic MS) shadow, and partially remyelinating plaques.

**Figure 2.**
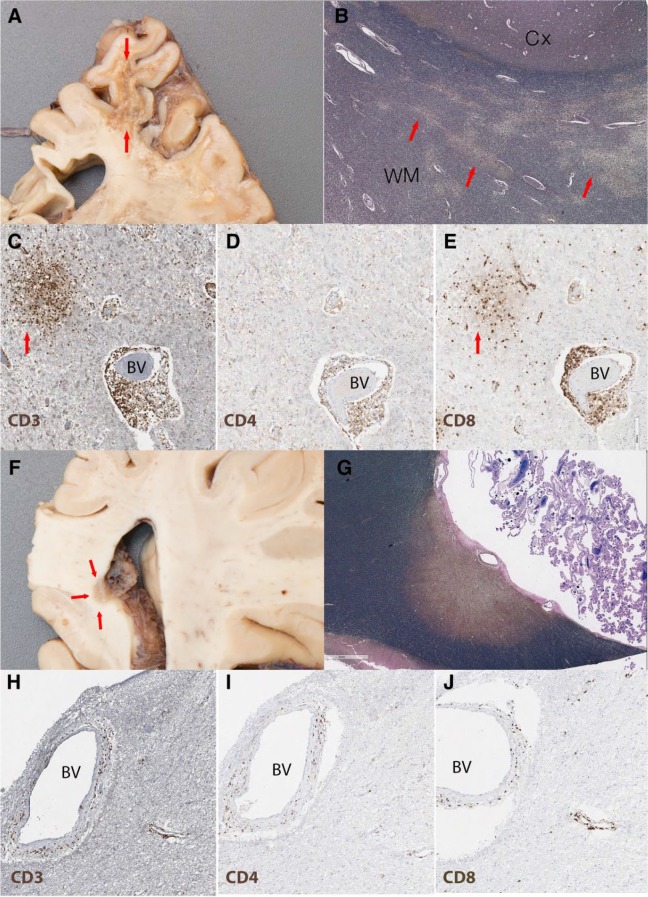
Gross and microscopic images of the PML chronic IRIS demyelinating lesions. (A-E) Chronic cavitary demyelinating plaque with high JC viral load (210 743 copies/10 mL DNA); (A) Cross section and high-power view of frontoparietal lesion with shrunken pitted white matter (arrows); (B) LFB/NF staining shows “salt on ice” pattern of demyelination (highlighted by arrows) in deep white matter (WM) and grey-white matter (Cx) junction; (C) CD3 staining (brown) highlights numerous T-cells around blood vessels (BV) but also in aggregates in the nearby parenchyma (arrow); (D) CD4 staining (brown) shows a subpopulation of T-cells; (E) CD8 staining (brown) demonstrates the CD8 predominance in both the perivascular and parenchymal (arrow) infiltrate. (F-J) Periventricular lesion in the splenium of the corpus callosum with low JC viral load (587 copies/10 mL DNA); (F) Coronal section trough the splenium of corpus callosum shows a well-demarcated periventricular demyelinating plaque (arrows); (G) LFB/NF staining shows a single well-demarcated chronic demyelinating periventricular plaque; the amount of inflammation is significantly less than the frontoparietal lesion in “C” with occasional perivascular T-cells. H, CD3; I, CD4; J, CD8. LFB/NF, Luxol fast blue (stains myelin blue)/neurofilament (stains axon black); CD3, T-cells, CD4 and CD8 subpopulation of T-cells.

A comparison between plaques from the frontoparietal region (PML by radiology) and a more confluent plaque in the periventricular posterior corpus callosum (MS-like) revealed evidence of ongoing PML-IRIS with different degrees of inflammatory infiltrate and JCV DNA copy numbers (Figure 2). The large frontoparietal cavitary demyelinating lesion (Figure 2A-E) contained a center with parenchymal loss, marked gliosis, chronic macrophage infiltrate, and axonal breakdown. At the white matter-grey matter junction, the lesion contained multiple microscopic demyelinating plaques with the “salt on ice” appearance (Figure 2B). Notably, there were multiple foci of perivascular mononuclear cells composed of a mixed population of lymphocytes, T-cell predominant with occasional B-cells, and no plasma cells (Figure 2C). T-lymphocytes were found in both perivascular and parenchymal regions with a preponderance of CD8^+^ as compared with CD4^+^ T-cells (Figure 2D and E), consistent with IRIS. In contrast, the number of inflammatory cells was significantly lower in the periventricular posterior corpus callosum (MS-like; Figure 2F-J). However, although they differed in abundance, the composition of infiltrates in the 2 regions was similar (Figure 2H-J).

CSF and formalin-fixed, paraffin-embedded tissue was sent to the NIH for JCV multiplex quantitative PCR analysis.^19^ Sections from the frontoparietal region contained 210 743 copies/10 mL DNA, while tissue from the corpus callosum contained 587 copies/10 mL DNA. The DNA sequence was consistent with the variant most commonly associated with PML. The CSF sample contained 54 copies/mL.

## Discussion

Our case is the first to demonstrate prolonged pathological IRIS with the continued perivascular inflammation 3.5 years after the PML diagnosis and 3 years after symptomatic stabilization. The imaging details the progression from PML to IRIS and eventually radiologic stabilization with extensive tissue destruction. There was also histopathological evidence of burned-out PML lesions, especially the demyelinating plaques that involve grey matter junction. The presence of perivascular mononuclear cells composed of T>B-lymphocytes and a CD8^+^ predominance is highly compatible with the neuropathologic description indicating active IRIS^20^ despite a negative SV40 stain, which was comparable to a previous report of IRIS lasting 9 months.^17^ Although there is approximately 70% sequence similarity between the DNA genomes of SV40 and JCV,^21^ they are distinct polyomaviruses; thus, a negative result for SV40 does not conclusively rule out the presence of JCV. Indeed, the more sensitive PCR analysis from formalin-fixed, paraffin-embedded tissue at the time of autopsy showed elevated JCV DNA copy numbers indicating that the initial infection never resolved, or that the patient’s immune system may have been compromised, allowing JCV to replicate more aggressively, which could be consistent with his development of urosepsis.

A clinical study of 336 patients examined the predictors of survival in natalizumab-associated PML patients and found 76% survival rate at 24 months.^16^ The survivors were significantly younger at diagnosis, had less disability prior to PML diagnosis, lower CSF JC viral load at the time of diagnosis, and more localized demyelinating plaques on MRI. Our patient’s age was above the average for survivors, but his low JCV viral load likely limited the virulence of PML. Additionally, his late development of IRIS as well as the treatments used to manage it, including maraviroc and cidofovir, may have led to his prolonged survival. In a study of 42 patients with natalizumab-related PML, those who developed early IRIS, about 3 weeks after PLEX, had significantly worse outcomes than those who developed late IRIS, about 4.5 weeks after PLEX.^5^ Our patient did not develop symptoms of IRIS until 7 weeks after PLEX.

The survival rate for patients with natalizumab-associated PML, which lack immunosurveillance specifically within the CNS, is significantly higher than for globally immunocompromised PML patient populations.^2^ These patients also have higher rates of PML-IRIS, suggesting that the ability to effectively reconstitute relevant lymphocyte populations within the CNS is a critical feature of survival. Our case study suggests that prolonged subclinical IRIS beyond clinical and radiological stabilization could also be related to longer survival. Since prolonged pathological IRIS can only currently be detected through biopsy, which is difficult to obtain from living patients, it would not have been incorporated into previous analyses of survival. An analysis of tissue biopsies from living PML patients with prolonged survival using immunohistochemistry to detect immune infiltrates and JCV multiplex PCR would be informative to address the prevalence of subclinical PML-IRIS. With the growing number of cases of natalizumab-associated PML in recent years, this type of analysis could help guide future treatment decisions. Unfortunately, this prolonged inflammation is likely a double-edged sword, as it could possibly contribute to the progressive brain atrophy detected by radiological and pathological analysis. Furthermore, it may also have resulted in immune exhaustion, and the eventual fatal outcome. There may, however, be a form of immune reconstitution that maximizes the clearance of JCV, while minimizing inflammatory damage that can be achieved through an optimized cocktail of drugs, perhaps including maravoric or other emerging therapies.^2^ Going forward peripheral immunological assays will be needed to help predict whether patients are likely to have productive or damaging forms of immune reconstitution, as well as to tailor the treatment regimen to produce an optimal response for each individual patient.
